# 67. Transitioning from Permissive to Restrictive Urine Reflex Criteria: Compiling the Data

**DOI:** 10.1093/ofid/ofab466.269

**Published:** 2021-12-04

**Authors:** Chad D Nix, Angela H Villamagna

**Affiliations:** 1 Oregon Health & Science University, Portland, Oregon; 2 Oregon Health and Science University, Portland, OR

## Abstract

**Background:**

Reflex urine cultures (UCx) are a diagnostic stewardship practice that limit the progression of UCx to specimens that meet pre-defined urinalysis criteria, but there is no widely recommended threshold for culture. At our institution, urinalyses (UAs) are reflexed to UCx for positive nitrites, leukocyte esterase, presence of bacteria, or ≥5 white blood cells per high powered field (WBC/hpf). Our aim is to assess if a more restrictive criteria of >10 WBC/hpf would result in missed UTI diagnoses.

**Methods:**

We performed a retrospective chart review of a systematic sampling of urine specimens collected from July 2018 to June 2019 in the emergency department and adult inpatient units. Inclusion criteria were UA with a WBC/hpf of 5-10 – samples that would not reflex to culture under our proposed criteria – and a UCx. We recorded signs, symptoms and antibiotic use via chart review. Positive UCxs were defined as ≥10e5 CFU/mL of bacterial growth (BG) and these cases were assessed using standardized CDC UTI definitions.

**Results:**

486 urine specimens with < 10e5 CFU/mL BG and 96 with ≥10e5 CFU/mL BG met inclusion criteria. Chart review was performed on 99 cases. 81 (82%) specimens had negative UCxs and 18 (18%) were positive. 45% had documented localizing UTI symptoms. 26% of all urine studies were sent for an indication of fever, 15% for altered mental status (AMS), and 8% for malaise. Among the 18 patients with positive UCxs, 11 (61%) met UTI criteria. Among the 81 patients with negative UCxs, 33/81 (41%) had a local symptom compatible with UTI. 7/81 (9%) patients had positive tests from other body sites; all 7 of these UCxs were sent for a new or worsening fever.

**Conclusion:**

Of the 99 UCxs reviewed, less than half had a urinary symptom consistent with UTI, and almost half of studies were sent for non-specific indications such as fever, which suggests reflex UCxs are overutilized at our institution. However, our data demonstrate that a more restrictive UCx criteria may not be the solution, as at least 11 clinically significant UTIs would have been missed under the new criteria. We recommend improved clinical decision support tools and more data to validate restrictive reflex UCx criteria before their implementation.

**Disclosures:**

**All Authors**: No reported disclosures

